# Extensive hydrogen-bonding network and an unusual cation conformation in [tris­(hydroxy­meth­yl)methyl]­ammonium tetra­oxidorhenate(VII)

**DOI:** 10.1107/S1600536809028797

**Published:** 2009-07-29

**Authors:** Małgorzata Hołyńska, Tadeusz Lis

**Affiliations:** a Faculty of Chemistry, University of Wrocław, 14 Joliot-Curie St, 50-383 Wrocław, Poland

## Abstract

The title compound, (C_4_H_12_NO_3_)[ReO_4_], contains two cations and two anions in the asymmetric unit, related by a non-crystallographic centre of symmetry. The crystal structure is stabilized by an extensive hydrogen-bonding network with the formation of puckered layers perpendicular to [001]. In the tris­(hydroxy­meth­yl)ammonium cations, intra­molecular O—H⋯O hydrogen bonds are present with the formation of an *S*_1_^1^(6) graph-set motif. The crystal structure is further consolid­ated by N—H⋯O hydrogen bonds.

## Related literature

For related structures, see: Castellari & Ottani (1997 ▶); Eilerman & Rudman (1980 ▶); Hołyńska & Lis (2004 ▶, 2008 ▶); Lock & Turner (1975 ▶); Marsh *et al.* (1998 ▶); Rudman *et al.* (1979 ▶, 1983 ▶); Shakked *et al.* (1980 ▶); Tusvik *et al.* (1999 ▶). For the dielectric properties of rhenates(VII) with organic ammonium cations, see: Czarnecki & Małuszyńska (2000 ▶). For graph-set notation, see: Etter *et al.* (1990 ▶). For the synthesis of rhenic(VII) acid, see: Johnson *et al.* (1967 ▶).
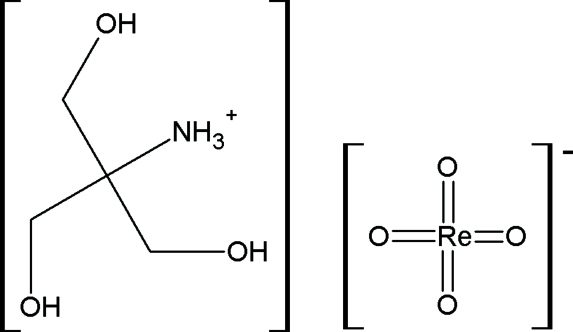


## Experimental

### 

#### Crystal data

(C_4_H_12_NO_3_)[ReO_4_]
*M* *_r_* = 372.35
Orthorhombic, 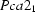

*a* = 21.450 (5) Å
*b* = 6.867 (2) Å
*c* = 12.219 (4) Å
*V* = 1799.8 (9) Å^3^
*Z* = 8
Mo *K*α radiation
μ = 13.51 mm^−1^
*T* = 110 K
0.21 × 0.16 × 0.14 mm

#### Data collection

Oxford Diffraction KM-4-CCD diffractometer
Absorption correction: analytical (**CrysAlis RED**; Oxford Diffraction, 2006 ▶) *T*_min_ = 0.104, *T*_max_ = 0.268
24604 measured reflections
5888 independent reflections
5084 reflections with *I* > 2σ(*I*)
*R*_int_ = 0.029

#### Refinement

*R*[*F* ^2^ > 2σ(*F* ^2^)] = 0.020
*wR*(*F* ^2^) = 0.034
*S* = 1.02
5888 reflections
245 parameters
1 restraint
H-atom parameters constrained
Δρ_max_ = 2.00 e Å^−3^
Δρ_min_ = −1.27 e Å^−3^

### 

Data collection: *CrysAlis CCD* (Oxford Diffraction, 2006 ▶); cell refinement: *CrysAlis RED* (Oxford Diffraction, 2006 ▶); data reduction: *CrysAlis RED*; program(s) used to solve structure: *SHELXS97* (Sheldrick, 2008 ▶); program(s) used to refine structure: *SHELXL97* (Sheldrick, 2008 ▶); molecular graphics: *DIAMOND* (Brandenburg & Putz, 2005 ▶) and *SHELXTL-NT* (Sheldrick, 2008 ▶); software used to prepare material for publication: *SHELXL97*.

## Supplementary Material

Crystal structure: contains datablocks I, global. DOI: 10.1107/S1600536809028797/ez2176sup1.cif

Structure factors: contains datablocks I. DOI: 10.1107/S1600536809028797/ez2176Isup2.hkl

Additional supplementary materials:  crystallographic information; 3D view; checkCIF report

## Figures and Tables

**Table:** 

Re1—O11	1.736 (2)
Re1—O21	1.728 (2)
Re1—O31	1.727 (2)
Re1—O41	1.702 (5)
Re2—O12	1.728 (4)
Re2—O22	1.730 (3)
Re2—O32	1.736 (3)
Re2—O42	1.726 (2)

**Table:** 

O41—Re1—O31	109.6 (2)
O41—Re1—O21	108.7 (2)
O31—Re1—O21	108.7 (2)
O41—Re1—O11	110.6 (2)
O31—Re1—O11	110.0 (2)
O21—Re1—O11	109.2 (2)
O42—Re2—O12	108.4 (2)
O42—Re2—O22	109.4 (2)
O12—Re2—O22	109.6 (2)
O42—Re2—O32	109.3 (2)
O12—Re2—O32	110.8 (2)
O22—Re2—O32	109.3 (2)

**Table 2:** Hydrogen-bond geometry (Å, °)

*D*—H⋯*A*	*D*—H	H⋯*A*	*D*⋯*A*	*D*—H⋯*A*
N2—H2*A*⋯O21	0.91	2.03	2.858 (4)	150
N2—H2*B*⋯O111^i^	0.91	1.88	2.788 (4)	173
N2—H2*C*⋯O31^ii^	0.91	1.98	2.879 (4)	169
O112—H112⋯O212	0.84	2.12	2.773 (4)	134
O112—H112⋯O41	0.84	2.49	2.942 (4)	115
O212—H212⋯O32^ii^	0.84	1.89	2.721 (4)	168
O312—H312⋯O112^ii^	0.84	1.92	2.704 (4)	156
N1—H1*A*⋯O312^iii^	0.91	1.83	2.738 (4)	176
N1—H1*B*⋯O42^ii^	0.91	2.05	2.872 (4)	150
N1—H1*C*⋯O22	0.91	1.98	2.862 (4)	164
O111—H111⋯O211^iv^	0.84	1.89	2.681 (3)	157
O211—H211⋯O311	0.84	2.13	2.774 (4)	134
O211—H211⋯O12^ii^	0.84	2.54	2.960 (4)	112
O311—H311⋯O11	0.84	1.88	2.714 (4)	170

Symmetry codes: (i) 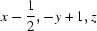; (ii) 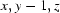; (iii) 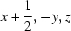; (iv) 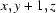.

**Fig. 1.**
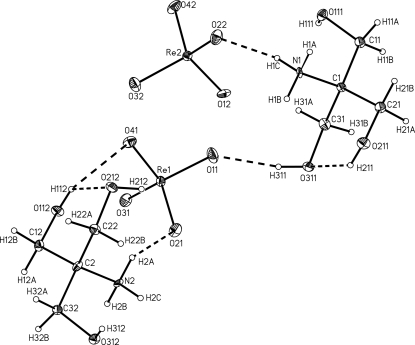
The symmetry-independent part of (1). Thermal ellipsoids are drawn at 30% probability level. Hydrogen bonds are denoted with dashed lines.

**Fig. 2.**
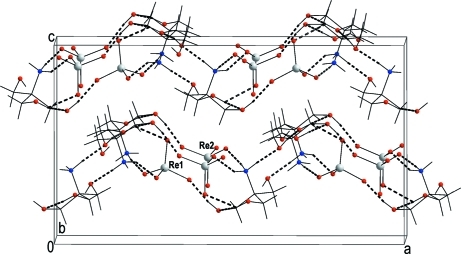
Puckered hydrogen-bonded layers perpendicular to [001]. Hydrogen bonds are denoted with dashed lines.

**Fig. 3.**
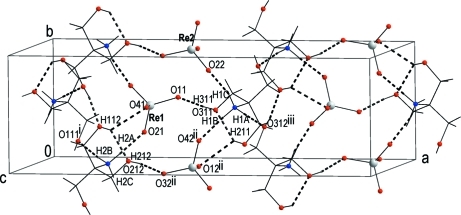
One of the layers with hydrogen bonding scheme. Hydrogen bonds are denoted with dashed lines. Symmetry codes as in Table 2.

## supplementary crystallographic information

### Comment

The title compound was obtained as starting material for other syntheses (*e.g.* reaction with acethyl chloride - Hołyńska & Lis, 2008). It was chosen as the tris(hydroxymethyl)methylammonium cation gives rise to an extensive hydrogen bonding network, which allows for selective crystallization of products containing Re, reducing the risk of cocrystallization of impurities and crystal structure disorder. Moreover, rhenates(VII) with organic ammonium cations crystallizing in non-centrosymmetric space groups are promising materials with respect to their dielectric properties. For example, the previously discovered ferroelectric with a Curie temperature above room temperature is pyridinium rhenate(VII) (Czarnecki & Małuszyńska, 2000).

The title compound (1) is a product of the reaction of rhenic(VII) acid with tris(hydroxymethyl)methylamine (TRIS) in aqueous solution, comprising discrete tris(hydroxymethyl)methylammonium cations (protonated TRIS here denoted as TRISH^+^) and rhenate(VII) anions (Fig. 1, Scheme 1).

There are two symmetry-independent rhenate(VII) anions (containing atoms Re1 and Re2, respectively) with the expected (see *e.g.* Hołyńska & Lis, 2004 for example of rhenate(VII) anions in low symmetry environment) slightly distorted tetrahedral geometry. The Re—O bond lengths are listed in Table 1. Their values are consistent with those for other rhenates(VII), *e.g.* 1.723 (4) Å for potassium rhenate(VII) reported by Lock & Turner (1975). These bond lengths are not much affected by the presence of hydrogen bonds, as all rhenate(VII) O atoms participate in these interactions as acceptors (Table 2).

It is interesting to note that both symmetry-independent TRISH^+^ cations are of unusual conformation. Usually the cation symmetry is close to C_3_ (*e.g.* Rudman *et al.*, 1983) or even exactly threefold (as in [TRISH]Cl appearing in a preliminary report by Rudman *et al.*, 1979) with no intramolecular hydrogen bonds. In (1) both cations exhibit the presence of such intramolecular hydrogen bond (Table 2) with the formation of a S_1_^1^(6) graph-set motif (Etter *et al.*, 1990). The relevant N—C—C—O torsion angles are given in Table 1. Bond lengths characterizing the cations, among them the C—N bond length (which is longer than in the TRIS molecule - 1.477 (3) Å as reported for the neutral TRIS molecule by Eilerman & Rudman, 1980) are in accordance with the values reported for other structures (*e.g.* Castellari & Ottani, 1997). TRIS is a constituent of buffers used in biochemical studies in the pH range of 7–9 (Castellari & Ottani, 1997). Upon protonation it forms salts with biologically relevant anions (*e.g.* tris(hydroxymethyl)methylammonium deoxycholate reported by Tusvik *et al.*, 1999), also a report on its interaction with nucleotides in the crystalline state is available (Shakked *et al.*, 1980).

All cation ammonium and hydroxyl groups are donors in N—H···O or O—H···O hydrogen bonds, both to other TRISH^+^ cations or to rhenate(VII) anions. The shortest Re···Re distance is 4.210 (2) Å. Thus, puckered hydrogen-bonded layers perpendicular to [001] are formed (Fig. 2). The hydrogen bonding scheme stabilizing an individual layer is illustrated in Fig. 3.

### Experimental

The title compound was obtained in the reaction of 0.19 g of tris(hydroxymethyl)methylamine (TRIS) with an excess of rhenic(VII) acid in aqueous solution, with slow evaporation leading to colourless crystals. The reaction was carried out in a quartz beaker. Rhenic(VII) acid was obtained according to the literature procedure (Johnson *et al.*, 1967) in reaction of 0.3 g of metallic Re with an excess of a 30% aqueous hydrogen peroxide solution.

### Refinement

The structure was solved by direct methods in the space group P1, and the present solution was obtained by switching to a higher symmetry. It was possible to end up in a false minimum in the Pca2_1_ space group (*e.g.* with the following approximate coordinates for the Re atoms: 0.57, 0.024, 0.97 for Re1; 0.69, 0.51, 1.01 for Re2; see Marsh *et al.*, 1998, for a review of some pitfalls connected with the Pca2_1_ space group). All H atoms were generated geometrically and refined with *U*_eq_=n*U*_eq_(parent atom), where n = 1.5 for hydroxyl H atoms, and n = 1.2 for the remaining H atoms. During the refinement, extinction was also taken into account. Furthermore, it was found that the structure is a racemic twin (with a refined BASF parameter value of 0.375 (6)). On the final difference Fourier map the highest peak of 2.00 e/Å^3^ was found at 0.62 Å from atom Re2. The crystal structure contains a pseudosymmetry centre at approximately (0.37, 0.73, 0.38).

### Crystal data

**Table:**

(C_4_H_12_NO_3_)[ReO_4_]	*F*(000) = 1392
*M**_r_* = 372.35	*D*_x_ = 2.748 Mg m^−^^3^
Orthorhombic, *P**c**a*2_1_	Mo *K*α radiation, λ = 0.71073 Å
Hall symbol: P 2c -2ac	Cell parameters from 20472 reflections
*a* = 21.450 (5) Å	θ = 4.2–35.0°
*b* = 6.867 (2) Å	µ = 13.51 mm^−^^1^
*c* = 12.219 (4) Å	*T* = 110 K
*V* = 1799.8 (9) Å^3^	Needle, colourless
*Z* = 8	0.21 × 0.16 × 0.14 mm

### Data collection

**Table:**

Oxford Diffraction KM-4-CCD diffractometer	5888 independent reflections
Radiation source: fine-focus sealed tube	5084 reflections with *I* > 2σ(*I*)
graphite	*R*_int_ = 0.029
ω scans	θ_max_ = 35.0°, θ_min_ = 4.2°
Absorption correction: analytical (*CrysAlis RED*; Oxford Diffraction, 2006)	*h* = −33→28
*T*_min_ = 0.104, *T*_max_ = 0.268	*k* = −9→11
24604 measured reflections	*l* = −14→19

### Refinement

**Table:**

Refinement on *F*^2^	Secondary atom site location: difference Fourier map
Least-squares matrix: full	Hydrogen site location: inferred from neighbouring sites
*R*[*F*^2^ > 2σ(*F*^2^)] = 0.020	H-atom parameters constrained
*wR*(*F*^2^) = 0.034	*w* = 1/[σ^2^(*F*_o_^2^) + (0.0119*P*)^2^] where *P* = (*F*_o_^2^ + 2*F*_c_^2^)/3
*S* = 1.02	(Δ/σ)_max_ = 0.002
5888 reflections	Δρ_max_ = 2.00 e Å^−^^3^
245 parameters	Δρ_min_ = −1.27 e Å^−^^3^
1 restraint	Extinction correction: *SHELXL97* (Sheldrick, 2008), Fc^*^=kFc[1+0.001xFc^2^λ^3^/sin(2θ)]^-1/4^
Primary atom site location: structure-invariant direct methods	Extinction coefficient: 0.00101 (3)

### Fractional atomic coordinates and isotropic or equivalent isotropic displacement parameters (Å^2^)

**Table:**

	*x*	*y*	*z*	*U*_iso_*/*U*_eq_	
Re1	0.311748 (6)	0.494021 (19)	0.362409 (13)	0.01257 (4)	
O11	0.38162 (12)	0.5562 (4)	0.2998 (2)	0.0266 (6)	
O21	0.29045 (12)	0.2623 (3)	0.3220 (2)	0.0217 (6)	
O31	0.25389 (12)	0.6547 (3)	0.3237 (2)	0.0204 (6)	
O41	0.31950 (13)	0.4971 (3)	0.5010 (4)	0.0192 (7)	
Re2	0.431704 (6)	0.975939 (17)	0.393938 (10)	0.01252 (4)	
O12	0.43101 (13)	0.9629 (4)	0.2527 (4)	0.0201 (7)	
O22	0.48425 (12)	0.8084 (4)	0.4456 (2)	0.0239 (6)	
O32	0.35819 (12)	0.9281 (4)	0.4468 (2)	0.0236 (6)	
O42	0.45440 (14)	1.2076 (3)	0.4316 (2)	0.0256 (6)	
N1	0.54415 (13)	0.4794 (3)	0.3475 (3)	0.0087 (7)	
H1A	0.5752	0.4330	0.3909	0.010*	
H1B	0.5116	0.3943	0.3478	0.010*	
H1C	0.5312	0.5969	0.3733	0.010*	
C1	0.56824 (16)	0.5031 (4)	0.2313 (5)	0.0108 (9)	
C11	0.62070 (17)	0.6555 (5)	0.2341 (3)	0.0158 (8)	
H11A	0.6583	0.5969	0.2674	0.019*	
H11B	0.6313	0.6942	0.1582	0.019*	
O111	0.60331 (12)	0.8244 (3)	0.2949 (2)	0.0161 (5)	
H111	0.5882	0.9079	0.2523	0.024*	
C21	0.59498 (17)	0.3079 (5)	0.1935 (3)	0.0161 (8)	
H21A	0.6055	0.3169	0.1148	0.019*	
H21B	0.6341	0.2820	0.2340	0.019*	
O211	0.55315 (12)	0.1482 (3)	0.2097 (2)	0.0190 (6)	
H211	0.5192	0.1715	0.1780	0.029*	
C31	0.51492 (18)	0.5698 (6)	0.1573 (3)	0.0171 (8)	
H31A	0.4977	0.6936	0.1858	0.020*	
H31B	0.5313	0.5947	0.0829	0.020*	
O311	0.46638 (12)	0.4291 (4)	0.1512 (2)	0.0194 (6)	
H311	0.4369	0.4628	0.1922	0.029*	
N2	0.19985 (14)	0.0038 (3)	0.4133 (3)	0.0106 (7)	
H2A	0.2325	0.0881	0.4100	0.013*	
H2B	0.1679	0.0501	0.3718	0.013*	
H2C	0.2120	−0.1145	0.3873	0.013*	
C2	0.17844 (17)	−0.0169 (5)	0.5312 (5)	0.0126 (9)	
C12	0.15266 (17)	0.1803 (5)	0.5682 (3)	0.0141 (7)	
H12A	0.1130	0.2060	0.5295	0.017*	
H12B	0.1436	0.1749	0.6476	0.017*	
O112	0.19500 (12)	0.3372 (3)	0.5474 (2)	0.0174 (6)	
H112	0.2293	0.3138	0.5778	0.026*	
C22	0.23218 (16)	−0.0833 (5)	0.6035 (3)	0.0150 (7)	
H22A	0.2164	−0.1076	0.6784	0.018*	
H22B	0.2489	−0.2075	0.5748	0.018*	
O212	0.28111 (11)	0.0562 (4)	0.6084 (2)	0.0205 (6)	
H212	0.3083	0.0288	0.5614	0.031*	
C32	0.12571 (16)	−0.1669 (5)	0.5331 (3)	0.0139 (7)	
H32A	0.1175	−0.2067	0.6096	0.017*	
H32B	0.0872	−0.1067	0.5038	0.017*	
O312	0.14087 (12)	−0.3355 (3)	0.4693 (2)	0.0149 (5)	
H312	0.1612	−0.4143	0.5080	0.022*	

### Atomic displacement parameters (Å^2^)

**Table:**

	*U*^11^	*U*^22^	*U*^33^	*U*^12^	*U*^13^	*U*^23^
Re1	0.01274 (6)	0.01028 (5)	0.01470 (9)	−0.00034 (4)	0.00299 (6)	0.00074 (6)
O11	0.0195 (15)	0.0232 (14)	0.0373 (17)	−0.0036 (11)	0.0120 (13)	−0.0001 (13)
O21	0.0299 (16)	0.0150 (12)	0.0201 (14)	−0.0036 (10)	0.0061 (12)	0.0004 (10)
O31	0.0252 (15)	0.0141 (12)	0.0218 (14)	0.0036 (10)	0.0011 (12)	−0.0006 (10)
O41	0.0268 (17)	0.0171 (14)	0.0137 (18)	−0.0031 (10)	−0.0014 (13)	0.0013 (11)
Re2	0.01365 (7)	0.01088 (5)	0.01303 (8)	−0.00034 (5)	0.00093 (6)	−0.00031 (8)
O12	0.0235 (16)	0.0188 (12)	0.0180 (18)	−0.0037 (11)	−0.0066 (12)	−0.0068 (13)
O22	0.0284 (16)	0.0237 (14)	0.0195 (14)	0.0086 (11)	0.0016 (12)	0.0010 (11)
O32	0.0176 (14)	0.0194 (13)	0.0336 (15)	−0.0005 (10)	0.0063 (11)	0.0011 (12)
O42	0.0393 (17)	0.0163 (12)	0.0210 (15)	−0.0084 (11)	0.0083 (12)	−0.0057 (10)
N1	0.0087 (13)	0.0032 (10)	0.014 (2)	−0.0006 (9)	0.0076 (14)	0.0021 (11)
C1	0.0109 (18)	0.0078 (15)	0.014 (3)	−0.0007 (13)	0.0004 (14)	0.0006 (11)
C11	0.0146 (19)	0.0104 (16)	0.022 (2)	−0.0019 (13)	0.0032 (15)	−0.0018 (14)
O111	0.0204 (14)	0.0121 (12)	0.0158 (13)	−0.0001 (10)	0.0032 (11)	−0.0008 (9)
C21	0.0166 (19)	0.0093 (16)	0.022 (2)	−0.0026 (13)	0.0030 (16)	−0.0016 (14)
O211	0.0208 (15)	0.0106 (11)	0.0256 (16)	−0.0020 (10)	−0.0012 (12)	0.0010 (11)
C31	0.016 (2)	0.0167 (19)	0.018 (2)	0.0009 (15)	0.0001 (15)	0.0032 (15)
O311	0.0127 (14)	0.0207 (14)	0.0250 (16)	−0.0001 (11)	−0.0028 (12)	−0.0077 (12)
N2	0.0144 (13)	0.0044 (12)	0.013 (2)	0.0016 (9)	−0.0018 (13)	−0.0049 (10)
C2	0.019 (2)	0.0092 (17)	0.010 (2)	0.0024 (12)	0.0001 (15)	−0.0013 (13)
C12	0.0154 (19)	0.0093 (15)	0.0176 (19)	0.0010 (12)	0.0031 (14)	−0.0003 (13)
O112	0.0152 (14)	0.0083 (11)	0.0287 (16)	−0.0024 (10)	−0.0020 (12)	−0.0010 (10)
C22	0.0139 (19)	0.0131 (16)	0.0182 (19)	0.0004 (12)	−0.0014 (14)	0.0007 (14)
O212	0.0124 (14)	0.0215 (13)	0.0276 (16)	−0.0009 (10)	−0.0023 (11)	−0.0056 (12)
C32	0.0139 (19)	0.0098 (16)	0.018 (2)	−0.0037 (12)	0.0023 (15)	−0.0022 (13)
O312	0.0173 (14)	0.0093 (11)	0.0182 (14)	0.0009 (9)	−0.0011 (11)	−0.0013 (9)

### Geometric parameters (Å, °)

**Table:**

Re1—O11	1.736 (2)	C31—O311	1.423 (4)
Re1—O21	1.728 (2)	C31—H31A	0.9900
Re1—O31	1.727 (2)	C31—H31B	0.9900
Re1—O41	1.702 (5)	O311—H311	0.8400
Re2—O12	1.728 (4)	N2—C2	1.519 (7)
Re2—O22	1.730 (3)	N2—H2A	0.9100
Re2—O32	1.736 (3)	N2—H2B	0.9100
Re2—O42	1.726 (2)	N2—H2C	0.9100
N1—C1	1.520 (7)	C2—C22	1.522 (6)
N1—H1A	0.9100	C2—C32	1.530 (5)
N1—H1B	0.9100	C2—C12	1.531 (5)
N1—H1C	0.9100	C12—O112	1.432 (4)
C1—C31	1.528 (6)	C12—H12A	0.9900
C1—C21	1.529 (5)	C12—H12B	0.9900
C1—C11	1.537 (4)	O112—H112	0.8400
C11—O111	1.427 (4)	C22—O212	1.422 (4)
C11—H11A	0.9900	C22—H22A	0.9900
C11—H11B	0.9900	C22—H22B	0.9900
O111—H111	0.8400	O212—H212	0.8400
C21—O211	1.431 (4)	C32—O312	1.433 (4)
C21—H21A	0.9900	C32—H32A	0.9900
C21—H21B	0.9900	C32—H32B	0.9900
O211—H211	0.8400	O312—H312	0.8400
			
O41—Re1—O31	109.6 (2)	O311—C31—H31A	109.2
O41—Re1—O21	108.7 (2)	C1—C31—H31A	109.2
O31—Re1—O21	108.7 (2)	O311—C31—H31B	109.2
O41—Re1—O11	110.6 (2)	C1—C31—H31B	109.2
O31—Re1—O11	110.0 (2)	H31A—C31—H31B	107.9
O21—Re1—O11	109.2 (2)	C31—O311—H311	109.5
O42—Re2—O12	108.4 (2)	C2—N2—H2A	109.5
O42—Re2—O22	109.4 (2)	C2—N2—H2B	109.5
O12—Re2—O22	109.6 (2)	H2A—N2—H2B	109.5
O42—Re2—O32	109.3 (2)	C2—N2—H2C	109.5
O12—Re2—O32	110.8 (2)	H2A—N2—H2C	109.5
O22—Re2—O32	109.3 (2)	H2B—N2—H2C	109.5
C1—N1—H1A	109.5	N2—C2—C22	110.5 (3)
C1—N1—H1B	109.5	N2—C2—C32	107.5 (4)
H1A—N1—H1B	109.5	C22—C2—C32	110.5 (3)
C1—N1—H1C	109.5	N2—C2—C12	107.8 (3)
H1A—N1—H1C	109.5	C22—C2—C12	111.5 (4)
H1B—N1—H1C	109.5	C32—C2—C12	108.9 (3)
N1—C1—C31	109.3 (3)	O112—C12—C2	112.6 (3)
N1—C1—C21	108.4 (3)	O112—C12—H12A	109.1
C31—C1—C21	111.4 (4)	C2—C12—H12A	109.1
N1—C1—C11	107.5 (4)	O112—C12—H12B	109.1
C31—C1—C11	110.9 (3)	C2—C12—H12B	109.1
C21—C1—C11	109.2 (3)	H12A—C12—H12B	107.8
O111—C11—C1	111.9 (3)	C12—O112—H112	109.5
O111—C11—H11A	109.2	O212—C22—C2	112.4 (3)
C1—C11—H11A	109.2	O212—C22—H22A	109.1
O111—C11—H11B	109.2	C2—C22—H22A	109.1
C1—C11—H11B	109.2	O212—C22—H22B	109.1
H11A—C11—H11B	107.9	C2—C22—H22B	109.1
C11—O111—H111	109.5	H22A—C22—H22B	107.8
O211—C21—C1	113.3 (3)	C22—O212—H212	109.5
O211—C21—H21A	108.9	O312—C32—C2	111.6 (3)
C1—C21—H21A	108.9	O312—C32—H32A	109.3
O211—C21—H21B	108.9	C2—C32—H32A	109.3
C1—C21—H21B	108.9	O312—C32—H32B	109.3
H21A—C21—H21B	107.7	C2—C32—H32B	109.3
C21—O211—H211	109.5	H32A—C32—H32B	108.0
O311—C31—C1	112.0 (3)	C32—O312—H312	109.5
			
N1—C1—C11—O111	−47.2 (4)	N2—C2—C12—O112	−51.4 (4)
N1—C1—C21—O211	51.0 (4)	N2—C2—C22—O212	64.0 (4)
N1—C1—C31—O311	−63.1 (4)	N2—C2—C32—O312	45.7 (4)

### Hydrogen-bond geometry (Å, °)

**Table:**

*D*—H···*A*	*D*—H	H···*A*	*D*···*A*	*D*—H···*A*
N2—H2A···O21	0.91	2.03	2.858 (4)	150
N2—H2B···O111^i^	0.91	1.88	2.788 (4)	173
N2—H2C···O31^ii^	0.91	1.98	2.879 (4)	169
O112—H112···O212	0.84	2.12	2.773 (4)	134
O112—H112···O41	0.84	2.49	2.942 (4)	115
O212—H212···O32^ii^	0.84	1.89	2.721 (4)	168
O312—H312···O112^ii^	0.84	1.92	2.704 (4)	156
N1—H1A···O312^iii^	0.91	1.83	2.738 (4)	176
N1—H1B···O42^ii^	0.91	2.05	2.872 (4)	150
N1—H1C···O22	0.91	1.98	2.862 (4)	164
O111—H111···O211^iv^	0.84	1.89	2.681 (3)	157
O211—H211···O311	0.84	2.13	2.774 (4)	134
O211—H211···O12^ii^	0.84	2.54	2.960 (4)	112
O311—H311···O11	0.84	1.88	2.714 (4)	170

Symmetry codes: (i) *x*−1/2, −*y*+1, *z*; (ii) *x*, *y*−1, *z*; (iii) *x*+1/2, −*y*, *z*; (iv) *x*, *y*+1, *z*.
